# Clinical validation of a population-based input function for 20-min dynamic whole-body ^18^F-FDG multiparametric PET imaging

**DOI:** 10.1186/s40658-022-00490-y

**Published:** 2022-09-08

**Authors:** André H. Dias, Anne M. Smith, Vijay Shah, David Pigg, Lars C. Gormsen, Ole L. Munk

**Affiliations:** 1 Department of Nuclear Medicine and PET Centre, Aarhus University Hospital, Palle Juul-Jensens Boulevard 165, 8200 Aarhus N, Denmark; 2 Siemens Medical Solutions USA, Inc., Knoxville, TN USA; 3 Department of Clinical Medicine, Aarhus University, Aarhus N, Denmark

**Keywords:** Dynamic whole-body PET, FDG, Parametric imaging, Patlak, Input function, PBIF, AIF

## Abstract

**Purpose:**

Contemporary PET/CT scanners can use 70-min dynamic whole-body (D-WB) PET to generate more quantitative information about FDG uptake than just the SUV by generating parametric images of FDG metabolic rate (MR_FDG_). The analysis requires the late (50–70 min) D-WB tissue data combined with the full (0–70 min) arterial input function (AIF). Our aim was to assess whether the use of a scaled population-based input function (sPBIF) obviates the need for the early D-WB PET acquisition and allows for a clinically feasible 20-min D-WB PET examination.

**Methods:**

A PBIF was calculated based on AIFs from 20 patients that were D-WB PET scanned for 120 min with simultaneous arterial blood sampling. MR_FDG_ imaging using PBIF requires that the area under the curve (AUC) of the sPBIF is equal to the AUC of the individual patient’s input function because sPBIF AUC bias translates into MR_FDG_ bias. Special patient characteristics could affect the shape of their AIF. Thus, we validated the use of PBIF in 171 patients that were divided into 12 subgroups according to the following characteristics: diabetes, cardiac ejection fraction, blood pressure, weight, eGFR and age. For each patient, the PBIF was scaled to the aorta image-derived input function (IDIF) to calculate a sPBIF, and the AUC bias was calculated.

**Results:**

We found excellent agreement between the AIF and IDIF at all times. For the clinical validation, the use of sPBIF led to an acceptable AUC bias of 1–5% in most subgroups except for patients with diabetes or patients with low eGFR, where the biases were marginally higher at 7%. Multiparametric MR_FDG_ images based on a short 20-min D-WB PET and sPBIF were visually indistinguishable from images produced by the full 70-min D-WB PET and individual IDIF.

**Conclusions:**

A short 20-min D-WB PET examination using PBIF can be used for multiparametric imaging without compromising the image quality or precision of MR_FDG_. The D-WB PET examination may therefore be used in clinical routine for a wide range of patients, potentially allowing for more precise quantification in e.g. treatment response imaging.

**Supplementary Information:**

The online version contains supplementary material available at 10.1186/s40658-022-00490-y.

## Introduction

Dynamic whole-body (D-WB) FDG PET/CT is a recently developed technique that allows for direct reconstruction of multiparametric images complementing the classic standard SUV image [1, 2]. Recent studies have evaluated the feasibility of this technique in a clinical setting, with further studies required to ascertain the clinical value of the technique [3–6]. However, this technique requires the use of dynamic PET acquisitions starting at injection time [7, 8], causing a severe limitation: the time patients must remain on the bed. Since these image acquisition times are at least 60 min, D-WB multiparametric protocols block precious camera time and can be uncomfortable for patients, severely limiting the clinical implementation of the technique. Shortening the required scan duration of D-WB FDG PET/CT imaging would therefore be of critical importance for its implementation in the clinic.

The direct reconstruction of WB multiparametric images is based on the linear Patlak analysis [9, 10], requiring late tissue time–activity curves (TACs) measured by PET and the use of a full input function (IF). The gold standard for calculating an IF is an arterial input function (AIF), obtained from serial arterial blood sampling. However, since this is not viable in a clinical setting, a validated alternative solution is to estimate the IF from the 60-min PET data using an image-derived input function (IDIF) [3, 5, 11]. Since the acquisition of the initial part of the scan after injection is required, some groups have proposed shorter “dual time-point” protocols [12, 13] where the IDIF is obtained from an initial 0–6 min scan over the heart region, and the patient is rescanned again later for example 60–75 min after injection. Although this alternative temporarily frees the scanner for other patients, it is logistically challenging and requires extra CT scans. A simpler alternative would be using a standardized IF obtained from population data, a so-called population-based input function (PBIF) [11, 14–16], which potentially eliminates the need to measure the early bolus portion of the IF. For each patient, the full IDIF is replaced by an individually scaled PBIF (sPBIF) using the aorta IDIF from the late D-WB PET passes. The required D-WB PET acquisition would then be shortened to only include the late passes needed for multiparametric reconstruction, with the total scan time that is very similar to today’s standard of care WB FDG acquisitions. The PBIF needs to be robust and ideally applicable for all patient groups. Reducing the scan time, for example, to a “short” 20-min PET scan protocol would make this technique tolerated by practically all patients and less prone to motion artefacts, while adding detailed information about the uptake of FDG in the different regions of the body.

The aims of this study were to: (1) Assess whether the gold standard AIF can be substituted by an aorta IDIF; (2) Calculate a 120-min FDG PBIF based on invasive arterial blood samples; (3) Evaluate whether an individual IDIF can be replaced by a sPBIF allowing for a clinically feasible 20-min D-WB PET scan protocol; and (4) Validate that the sPBIF yields qualitatively and quantitatively acceptable parametric images in a large cohort of clinical patients.

## Methods

### Patient population

The study subjects were recruited from the department’s cohort of patients referred for FDG PET/CT as part of their clinical diagnostic work-up or treatment response evaluation. Therefore, this population reflects a randomized mixture of adult oncological patients undergoing routine PET at a nuclear medicine department. The study was approved by the local ethics committee in Region Midtjylland (1-10-72-223-19).

There were two study cohorts. The AIF group is a cohort of 20 individuals (11 female, 9 male) where we performed arterial blood sampling during a 120-min D-WB PET scan. The AIF group’s indications for PET referral, sex and age are shown in Table 1. Inclusion into this group was solely based on whether patients were deemed fit to lie still for 120 min while in the PET/CT scanner and agreed to an insertion of an arterial catheter. Data from the AIF Group were used to generate the optimal PBIF using the arterial blood sampling as the gold standard. The data from this group were excluded from further analysis so as to not bias the validation of the PBIF results.

**Table 1:** AIF group: PET study indication and population demographics

	Number of patients			Age distribution*		
Scan indication	Total	Male	Female	Total	Male	Female
Breast cancer	1	0	1	62	–	62
Cancer of unknown primary origin	1	0	1	58	–	58
Gastro-intestinal cancer	3	0	3	57 [49–66]	–	57 [49–66]
Gynaecological cancer	2	0	2	65.5 [58–73]	–	65.5 [58–73]
Infection & inflammation	3	2	1	51.7 [30–67]	62.5 [58–67]	30
Lung cancer	4	3	1	69.8 [56–78]	67 [56–76]	78
Lymphoma	6	4	2	56.3 [29–77]	62 [46–77]	45 [29–61]
Total	20	9	11	59.7 [29–78]	63.8 [46–77]	56.4 [29–78]

*values presented as mean [range]

A second cohort was the Test Group that was used for a retrospective analysis of the performance of the PBIF in patients with a set of characteristics that we consider could hinder the applicability of a generic PBIF. This cohort of 171 unique patients had 70-min D-WB scans and was divided into 12 sub-groups, paired into six “test” versus “control” groups according to the following characteristics: Patients with diabetes (*N* = 20) vs patients without diabetes (*N* = 20); patients with low cardiac ejection fraction (EF), defined by EF < 50% measured on echocardiography performed by a cardiologist (*N* = 11) vs patients with cardiac EF > 60% (*N* = 20); patients with elevated blood pressure, defined by BP > 140 (*N* = 20) vs patients with BP < 120/80 (*N* = 20); patients with excess weight, defined by BMI > 30 (*N* = 20) versus patients with normal BMI, defined by BMI 20–22 (*N* = 20); younger patients, defined by age < 38 (*N* = 20) vs older patients, defined by age > 75 (*N* = 20); and finally patients with low eGFR, defined by eGFR < 60 (*N* = 20) vs patients with normal eGFR, defined by eGFR > 90 (*N* = 20). As a selection factor for group division, no patient in one of the “test” groups could be a part of any “control” groups, but patients could be a part of multiple “test” or “control” groups simultaneously. Exempt from this rule were of course the age subgroups. The 12 sub-groups indications for PET referral, sex and age are shown in Additional file 1: Tables S1(A–F).

### Dynamic whole-body PET (D-WB PET)

All patients were scanned using a fully automated multiparametric D-WB PET acquisition protocol on a Siemens Biograph Vision 600 PET/CT scanner (Siemens Healthineers, Knoxville, TN, USA) with 26.2 cm axial field-of-view as described in Dias et al. [5]. Blood glucose level and blood pressure were measured in all patients.

An Intego PET Infusion System (MEDRAD, Inc., Warrendale, PA, USA) was used for standardized tracer infusions through a peripheral venous catheter in the patient’s arm [17]. The ^18^FDG was infused (0.1–3.0 mL; usually 0.1 mL) at PET scan start and flushed with saline (total volume of ^18^FDG solution and saline is 35 mL). The injection speed was 1 mL/sec and the injection lasted approximately 35 s. See Additional file 1: Fig. S1 for details about the shape of the injected bolus.

First, a low-dose WB CT (25 Ref mAs, 120 kV, Care Dose4D, Care kV, Admire level 3) was performed. Then, the 70-min multiparametric PET acquisition protocol was started at the time of injection of FDG (4 MBq/kg). This protocol consisted of (1) a 6-min dynamic scan with the bed fixed at the chest region including aorta (26 time frames: 12 × 5 s, 6 × 10 s, 8 × 30 s), and (2) a 64-min dynamic WB PET scan consisting of 16 continuous bed motion passes: 7 × 2 min WB passes followed by 9 × 5 min WB passes. The total PET scan time including bed motion between the craniocaudal WB passes was around 70 min. Finally, the patient had a super-low-dose WB CT (8 Ref mAs, 120 kV, Care Dose4D, Care kV, Admire level 3).

**Fig. 1:**
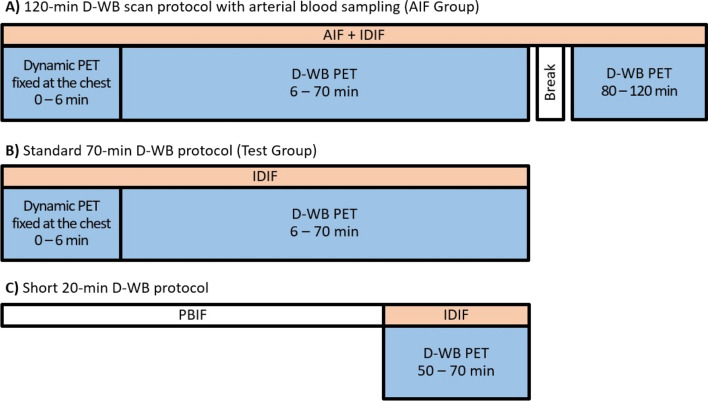
D-WB PET scan protocols used in the study + the input functions used for multiparametric imaging: **A** 120-min D-WB PET scan protocol with simultaneous arterial blood sampling used for the AIF Group; **B** Standard 70-min D-WB PET scan protocol used for the Test Group; **C** Short 20-min D-WB PET scan protocol using PBIF. The patients also had low-dose CT scans (not shown)

Patients in the AIF Group (*N* = 20) had a short break, an additional super-low dose CT, and 8 × 5 min WB from 80 to 120 min. The D-WB PET data acquisition protocols are shown in Fig. 1.

The PET reconstruction parameters for D-WB: For the SUV image, we used TrueX + TOF, 6 iterations, 5 subsets, 440 × 440 matrix, no filtering, and relative scatter correction. For the dynamic PET images used for IDIF extraction, we used TrueX + TOF, 4 iterations, 5 subsets, 440 × 440 matrix, no filtering, and relative scatter correction. Parametric images of MR_FDG_ and DV_FDG_ were generated using the nested direct Patlak reconstruction method with non-negativity constraints using list-mode data from four 5 min passes (50–70 min), TrueX + TOF, 8 iterations, 5 subsets, 30 nested loops, 440 × 440 matrix, 2-mm Gaussian filter, and relative scatter correction.

### Multiparametric imaging

In this paper, we focus on multiparametric imaging using the irreversible Patlak model [9]

1 $$C_{{\text{t}}} \left( t \right) = K_{i} \mathop \smallint \limits_{0}^{t} C_{{\text{a}}} \left( {t^{\prime } } \right){\text{d}}t^{\prime } + {\text{DV}} C_{{\text{a}}} \left( t \right),\;\;t > t^{*}$$

where *C*_t_ is the tracer activity concentration in tissue and *C*_a_ is the tracer concentration in arterial blood, i.e. the input function supplying the tissue. The parameters are the slope (*K*_*i*_), which is the rate of irreversible uptake, and the Patlak intercept (DV), which is the apparent distribution volume of non-metabolized tracer. The metabolic rate of FDG is MR_FDG_ = *K*_*i*_ × glc, where glc is the subject’s measured blood glucose. The Patlak model assumes irreversible uptake of the tracer and is valid after the non-metabolized tracer has reached a steady-state between blood and tissue, which is indicated by the time t^*^ in Eq. 1 that depends on the physiological properties of the tissue. Thus, the estimation of MR_FDG_ and DV requires a complete arterial input function (*C*_a_ at all times), but only late tissue data (*C*_t_ after t^*^).

Multiparametric Patlak images can be generated using direct reconstruction of parametric images or indirect image-based methods, and the direct methods have been shown to have favourable bias and noise characteristics [18]. In general, the noise and bias in *K*_*i*_ images depend on the specific implementation of the optimization algorithm, the mathematical formulations of the Patlak model, and the use of (non-negativity) constraints [16, 19]. This should be taken into account when comparing *K*_*i*_ values from different papers. In this paper, we used the direct Patlak reconstruction with nested loops and non-negativity constraints [20],which is available on Siemens PET/CT scanners. MR_FDG_ values obtained by this implementation has recently been compared to MR_FDG_ values obtained by the traditional image-based method [6].

We aim to shorten the multiparametric D-WB PET scan protocol and only scan a short series of late passes. This requires a PBIF that can be scaled to late IDIF data and provide the correct estimate of AUC up to scan start. As seen in the Patlak equation (Eq. 1), any bias on the AUC directly affects the estimate of *K*_*i*_. Furthermore, a recent study demonstrated that the error caused by the use of a PBIF with a late dominant single exponential after t* affects the global scale of the *K*_*i*_ image, and that the error is proportional to the AUC error [16]. PBIFs can be used as input function for any Patlak analysis method, and we focus on the effects of using PBIF independent of a specific Patlak analysis implementation by reporting AUC errors.

### Image-derived input function (IDIF)

Siemens Healthineers has developed an automated finding of anatomical landmarks on CT images, using a technology known as automated learning and parsing of human anatomy (ALPHA) [21]. The IDIF can be automatically generated from both the left ventricular blood pool (LV) and proximal descending aorta using ALPHA. For this study, two IDIFs were generated from using the aorta landmark found by ALPHA using different shaped VOIs (Additional file 1: Fig. S2): a cylinder-VOI of length 20 mm and radius 5 mm (volume 1.6 cm^3^) and a snake-VOI that was approximately the same volume but was only one voxel in width and several voxels long, forming a “snakelike” VOI shape (volume 0.9 cm^3^). These VOIs used to extract the IDIFs were automatically placed on the low dose CT. The method relies on good alignment between CT and PET, and we carefully checked the placement of the cylinder-VOI and snake-VOI in the aorta.

### Arterial input function (AIF) by invasive blood sampling

The 20 patients in the AIF Group had an arterial line placed for blood sampling. An automatic Allogg ABSS blood sampler (Allogg, Sweden) was used for the first 5 min (Pump speed: 7 mL/min; 1 sample/sec; tube setup: catheter + 3-way stopcock + 1-way stopcock + 25.5 cm tube with 1.65 mm inner diameter, total volume 1.22 mL). Then, the Allogg ABSS was stopped and the 1-way stopcock was disconnected from the patient.

Manual blood samples (catheter + 3-way stopcock, total volume 0.36 mL) were withdrawn prior to the WB passes, once for each of the 16 WB passes when the chest region is inside the PET detector, and finally after the PET scan; in total 18 manual blood samples from 6 to 70 min. After a short break, the patients had an additional D-WB scan from 80 to 120 min with one blood sample before the scan, once for each of the 8 WB passes, and one after the scan. In total, 18 + 10 = 28 manual blood samples from 6 to 120 min. Prior to each 1 mL manual blood sample, excess blood was withdrawn to ensure that the arterial line was filled with new blood. Test tubes were filled with 200 μL blood, and all manual whole blood samples were counted in a Hidex AMG (Hidex, Finland) well counter.

The automatic blood samples were corrected for dispersion [22] using the parameters: *α *= 0.5 and *k* = 5.6 min^−1^ that were measured in a separate experiment (data not shown). In addition, the blood time–activity curves were time-shifted to correct for the 10.5-s time delay caused by the 1.22 mL external tubes. The initial 0–5 min automated blood TAC was attached with the late 6–120 min manual blood TAC to obtain a combined 120-min dispersion- and delay-corrected arterial blood TAC.

Finally, this TAC was time-shifted to match the IDIF to correct for the internal time delay between the descending aorta, where the IDIF is extracted, and the arterial blood sampling site. This invasively measured arterial blood input function is denoted AIF for the remainder of the paper.

The PET scanner and blood sampling equipment were cross-calibrated to well within 5% using ^68^Ge/^68^ Ga solutions as part of our QC procedures.

### Population-based input function (PBIF)

The PBIF represents the standard shape of the AIF following injection of ^18^FDG. First, each AIF was normalized to its own 60-min AUC to remove differences in amplitude and to focus on the shape. Specifically, the AUC was calculated from t_0_ to 60 + t_0_ min, where t_0_ is the time of the initial rise of the AIF. These normalized AIFs were used generate the PBIF.

This PBIF shape is influenced by the shape of the tracer bolus that is administrated to the patient, and by the blood-tissue exchange of tracer in the patient’s body after injection. Thus, the shape of the PBIF is calculated as

2 $$C_{{{\text{PBIF}}}} \left( t \right) = B\left( t \right) \otimes h\left( t \right)$$

where *B(t)* is the bolus shape of the tracer infusion, $\otimes$ denotes convolution, and *h(t)* is the impulse-response function of the tracer circulating in blood.

For all patients, we used a standardized tracer infusion, and we have measured the normalized shape *B(t)* as shown in Additional file 1: Fig. S1. The impulse-response function of the tracer in blood, *h(t)*, was modelled with two multiexponential impulse-response functions *h(t)*. First, a three-exponential function *h*_*3*_*(t)* that describes the impulse-response function of a tracer in a circulatory system [23], which have been used recently to model arterial input functions [11].

3 $$h_{3} \left( t \right) = \left\{ {\begin{array}{*{20}l} {0,} \hfill & {t < \tau } \hfill \\ {\left[ {A_{1} \left( {t - \tau } \right) - A_{2} - A_{3} } \right]e^{{ - \lambda_{1} \left( {t - \tau } \right)}} + A_{2} e^{{ - \lambda_{2} \left( {t - \tau } \right)}} + A_{3} e^{{ - \lambda_{3} \left( {t - \tau } \right)}} ,} \hfill & { t \ge \tau } \hfill \\ \end{array} } \right.$$

Second, a model with an additional exponential *h*_*4*_*(t)* that potentially could provide better fits to the late data.

4 $$h_{4} \left( t \right) = \left\{ {\begin{array}{*{20}l} {0,} \hfill & {t < \tau } \hfill \\ {\left[ {A_{1} \left( {t - \tau } \right) - A_{2} - A_{3} - A_{4} } \right]e^{{ - \lambda_{1} \left( {t - \tau } \right)}} + A_{2} e^{{ - \lambda_{2} \left( {t - \tau } \right)}} + A_{3} e^{{ - \lambda_{3} \left( {t - \tau } \right)}} + A_{4} e^{{ - \lambda_{4} \left( {t - \tau } \right)}} ,} \hfill & {t \ge \tau } \hfill \\ \end{array} } \right.$$

*A*_1–4_ are the coefficients, *λ*_1–4_ are the eigenvalues, and τ is the time delay.

The PBIF was generated in three steps: (1) The PBIF model was fitted to each AUC_60_-normalized AIF using nonlinear regression; (2) Each fitted normalized AIF was corrected for its individual time delay parameter *τ* and averaged; (3) The PBIF model was fitted to the averaged normalized AIF to obtain the final set of PBIF parameters. The three-step procedure was repeated for *h*_*3*_*(t)* and *h*_*4*_*(t)*, and using 70 min AIF data and 120 min AIF data. During fitting, the data were weighted proportional to the time between measurements.

### Individualized scaled PBIF (sPBIF)

The PBIF is an estimate of the standard shape of the ^18^FDG blood input function. The PBIF is individualized by scaling the PBIF to the patient’s aorta IDIF as measured on the late passes used for multiparametric imaging. This sPBIF accounts for variations in injected dose, the patient’s body composition and tracer distribution. Three methods were tested to scale the PBIF to the patient’s own IDIF data from four D-WB passes from 50 to 70 min: Method 1: Mean value of the four IDIF values divided by the corresponding four interpolated PBIF values. This method did not require curve fitting. Method 2: AUC of a mono-exponential function fitted to the four IDIF points divided by the AUC of the PBIF. This model used a fit with two free parameters to four data points. Method 3: AUC of a constrained mono-exponential function fitted to the four IDIF points: First, a mono-exponential function was fitted to the PBIF within the time interval of the four IDIF points; Then, the estimated PBIF decay constant was used as a fixed parameter in the mono-exponential fit to the four IDIF points. This model used a fit with one free parameter to four data points.

### Selecting and validating the PBIF

Different shapes of the PBIF can lead to bias on the AUC of the sPBIF, and according to Eq. 1, this will translate into bias on the estimated metabolic rate MR_FDG_. Ideally, a (“perfectly shaped”) PBIF should lead to small error, preferably no error, when scaled to the subject’s IDIF. We tested the use of sPBIF instead of AIF for subjects in the AIF Group. We simulated different 20-min D-WB PET scan start times: 30–50 min, 40–60 min, 50–70 min, 80–100 min, 90–110 min, 100–120 min.

For the clinical validation, we calculated the sPBIF AUC bias in 171 patients that were not used to generate the PBIF. Special patient characteristics could affect the shape of their AIF. Thus, the 171 patients were divided into 12 subgroups according to the following characteristics: diabetes, cardiac ejection fraction, blood pressure, weight, eGFR and age.

### Whole blood IF vs plasma IF

AIF and IDIF were measured in whole blood, which makes them directly comparable. This way, the PBIF is created based on whole blood AIF measurements, and the PBIF can be correctly scaled to the patient’s late IDIF measurements to obtain the individualized whole blood sPBIF. Thus, *K*_*i*_ should be interpreted as the rate of irreversible uptake from FDG in whole blood to the trapped FDG-6P in tissue.

Ideally, kinetic modelling should involve both the arterial whole blood IF that describes the activity concentration in the blood volume, and the metabolite-corrected arterial plasma IF that describe the activity concentration available for uptake in tissue. Fortunately, for FDG, there are no blood-borne metabolites, and there is a relatively rapid exchange between plasma and red blood cells, where the whole blood to plasma ratio can be described by a mono-exponential function [11]. If needed, the *K*_*i*_ and MR_FDG_ estimates, which are based on a whole blood sPBIF, could be corrected by a small factor that accounts for the plasma AUC being around 3% higher than the whole blood AUC. Thus, *K*_*i*_ and MR_FDG_ estimates based on a plasma IF would be around 3% lower. We did not apply such a correction.

### Statistical analysis

All results are given as mean ± standard deviation (SD). Correlations were assessed by Pearson *r*. All analyses were made using IDL 8.0 (ITT Visual Information Solutions, Boulder, CO).

## Results

### *PBIF generation from arterial blood samples: AIF Group (N* = *20)*

The final parameters describing the PBIFs are shown in Table 2. The lower AIC numbers indicate that the four-exponential model (*h*_*4*_) provided better fits to the 20 invasively measured AIFs from the AIF Group both when fitting AIF data up to 70 min, and when fitting AIF data up to 120 min. The larger and more flexible model led to a lower final dominant exponential (*λ*_3_ for *h*_*3*_ and *λ*_4_ for *h*_*4*_).

**Table 2:** Final PBIF parameters estimates

Model	*h*_*3*_	*h*_*4*_	*h*_*3*_	*h*_*4*_
Time period fitted	0–70 min	0–70 min	0–120 min	0–120 min
*τ* (min)	0.15	0.16	0.15	0.16
*A*_1_ (min^−1^)	10.5	12.5	10.3	11.1
*λ*_1_ (min^−1^)	14.1	16.8	13.8	14.7
*A*_2_	0.029	0.046	0.031	0.022
*λ*_2_ (min^−1^)	0.28	2.56	0.23	0.53
*A*_3_	0.021	0.024	0.018	0.016
*λ*_3_ (min^−1^)	0.013	0.17	0.010	0.089
*A*_4_	–	0.018	–	0.016
*λ*_4_ (min^−1^)	–	0.011	–	0.0085
AIC	2102 ± 127	2082 ± 119	2014 ± 123	1989 ± 121

AIC results are mean ± SD (*N* = 20). The last column shows parameters for the reference PBIF that produced lowest AIC

**Fig. 2:**
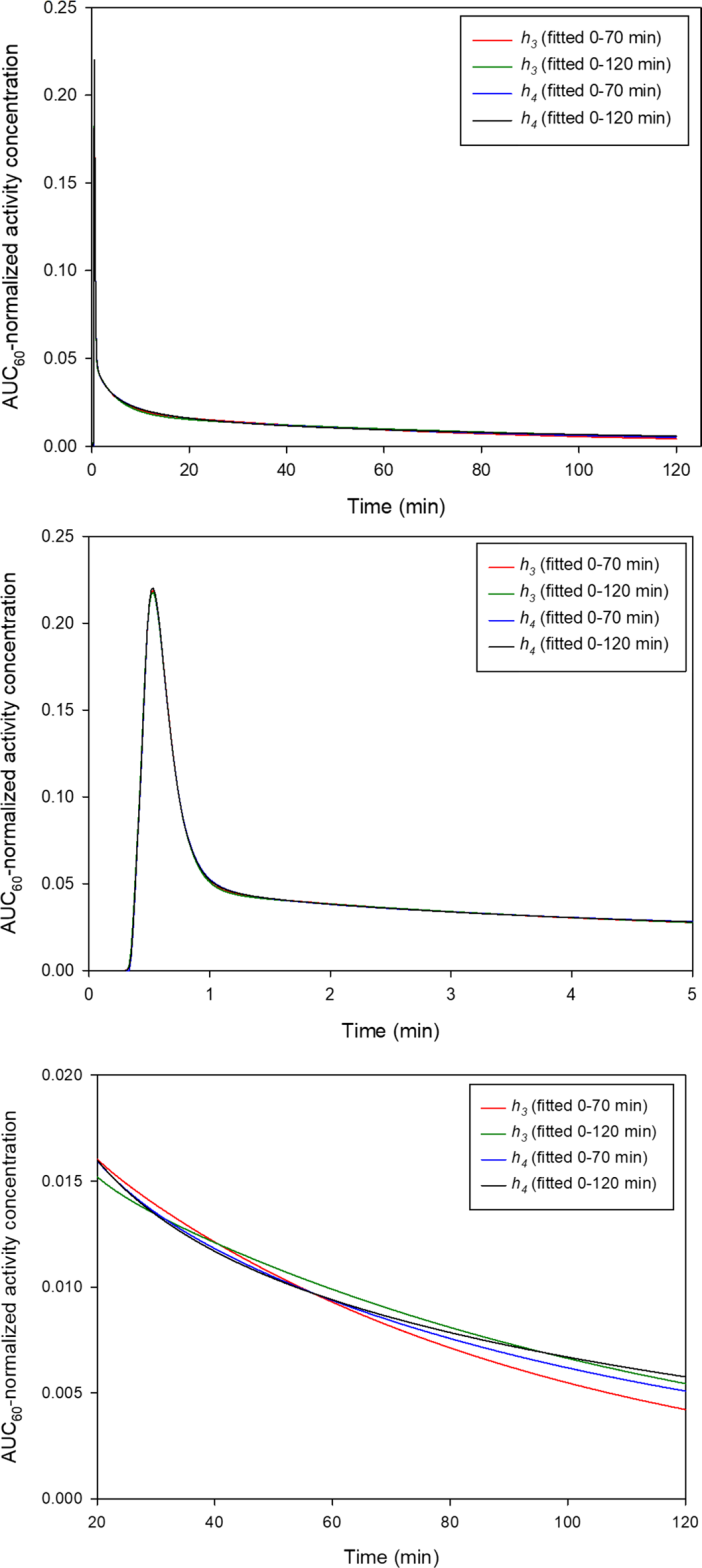
Three plots of the four PBIF shown at different time scales. Top: The four PBIFs are look almost identical on the full time-scale up to 120 min. Middle: The PBIFs have completely overlapping shapes of initial dynamic phase following the tracer infusion as modelled with the first exponentials. Bottom: After 20 min, the shapes are markedly different as modelled by the late exponentials

Four PBIFs were generated using the parameters in Table 2. The initial shapes of the four PBIFs were almost indistinguishable, whereas the differences became more pronounced after 10 min as seen both on the time courses for the PBIFs (Fig. 2) and their AUCs (Additional file 1: Fig. S3). The different shapes of the four PBIFs led to bias on the AUC of the sPBIF, which will directly affect the MR_FDG_ estimate (Eq. 1). This was explored in the AIF Group by scaling (Method 1) the four PBIFs to 20 min AIF at 6 different time points that simulate different start times for a 20 min D-WB parametric scan protocol: 30–50 min, 40–60 min, 50–70 min, 80–100 min, 90–110 min, 100–120 min.

**Fig. 3:**
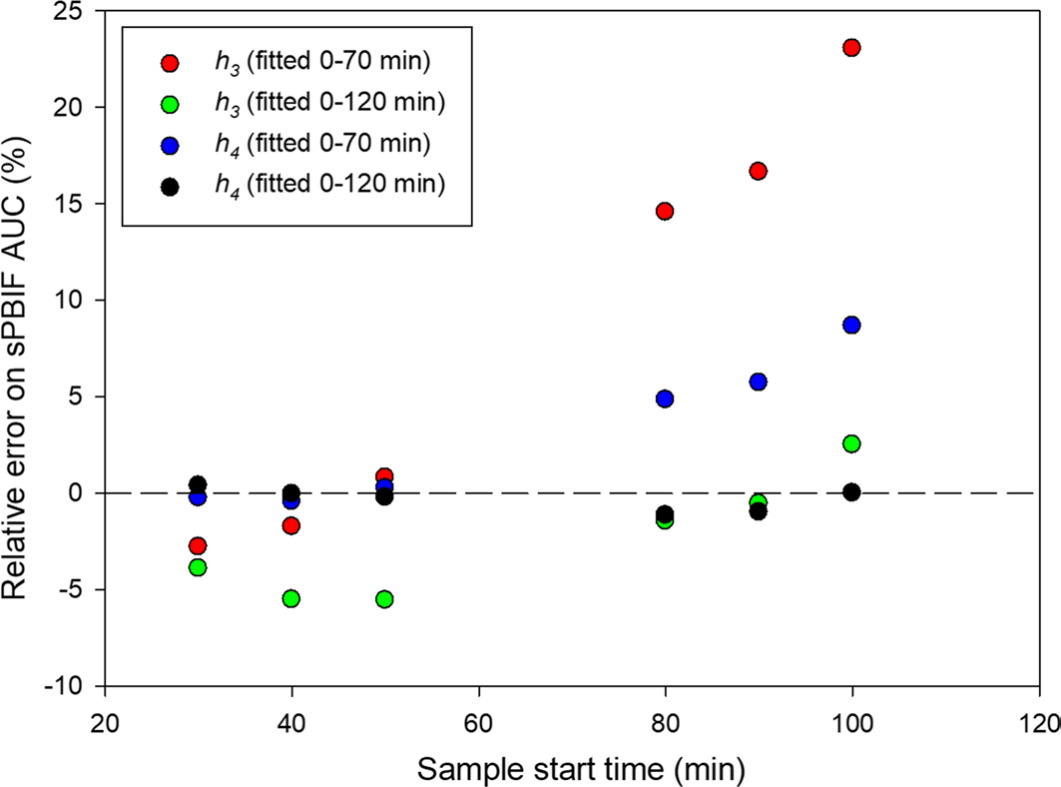
Error AUC of sPBIF relative to AUC of AIF. The sPBIF was scaled using four blood samples with 5 min interval and scaling method 1. The relative errors are shown as a function of the sampling time of the 1st blood sample, and AUCs are calculated up to the sampling time of the 4th blood sample, e.g. for the data points at 30 min, we scaled the PBIF using blood samples at 30 min, 35 min, 40 min and 45 min, and the AUCs are calculated up to 45 min. Clearly, the best PBIF is based on h_4_ and the use of data up to 120 min, as seen by the low AUC errors within 1%, i.e. best MR_FDG_ estimates

Based on these results, shown in Table 3 and Fig. 3, we proceed with the PBIF based on *h*_*4*_ (Eq. 4) and fitted to data up to 120 min (Table 2, last column). The results show the importance of using a PBIF based on a sufficiently complex model and many data points. The use of AIF data up to 120 min ensures that the PBIF can be used for Patlak imaging at any time up to 2 h p.i. without introducing additional bias. This reference PBIF had the best performance with an AUC error within 1% up to 120 min, and it is available for download as Additional files 2 and 3.

**Table 3:** Errors on AUC of sPBIF relative to AIF at different time points

Model	*h*_*3*_	*h*_*4*_	*h*_*3*_	*h*_*4*_
Time period fitted	0–70 min	0–70 min	0–120 min	0–120 min
Simulated scan time				
30–50 min	− 3% ± 6%	0% ± 6%	− 4% ± 6%	0% ± 6%
40–60 min	− 2% ± 7%	0% ± 7%	− 6% ± 6%	0% ± 7%
50–70 min	1% ± 8%	0% ± 8%	− 6% ± 7%	0% ± 8%
80–100 min	15% ± 11%	5% ± 10%	− 2% ± 9%	− 1% ± 9%
90–110 min	17% ± 11%	6% ± 10%	− 1% ± 9%	− 1% ± 9%
100–120 min	23% ± 12%	9% ± 10%	3% ± 10%	0% ± 10%

Values are mean ± SD (*N* = 20). The last column shows parameters for the reference PBIF that produced lowest errors. Each row represents the results for each different simulated 20 min D-WB PET scan start time

### *Robustness of automatic IDIF extraction: All patients (N* = *191)*

We extracted snake-VOIs and cylinder-VOIs on all patients using the low dose CT images (an example of these VOIs is shown in Additional file 1: Fig. S2). In these patients, both methods failed once, i.e. both methods successfully generated a VOI in the descending aorta in more than 99% of the cases. The single patient where the automatic VOI generation failed had to be scanned with one arm on the chest. In the remaining 190 cases, visual inspection showed that snake-VOIs was always well-centred, whereas the cylinder-VOI was slightly off-centre in 9 cases (3 needed manual adjustment to keep the VOI within aorta; an example is shown in Additional file 1: Fig. S4). Thus, both methods were very robust, but the cylinder-VOI occasionally needed manual repositioning due to its larger diameter.

### *Comparison of IDIFs with invasive AIF: AIF group (N* = *20)*

Visual inspection of the measurements showed excellent agreement between AIF and IDIFs both around the early peak and the late data. The correlations between the IDIFs and AIF were (Pearson correlation coefficient as mean ± SD): IDIF_Snake_ 0.992 ± 0.004; and IDIF_Cyl_ 0.988 ± 0.007. As discussed in the Methods section, the IF data needed for Patlak modelling are the IF AUC up to scan start and the IF measurements during scanning. The errors on AUC (0–60 min) for IDIFs relative to AIF were IDIF_Snake_ 3% ± 6% and IDIF_Cyl_ 3% ± 4%. The errors on the AUC (4 late passes: 50–70 min) for IDIFs relative to AIF were IDIF_Snake_ 3% ± 8% and IDIF_Cyl_ 4% ± 6%.

### *Comparison of PBIF scaling methods: AIF group (N* = *20)*

The three scaling methods were explored in the AIF group using the reference PBIF (Table 2, last column). For each subject, the scale factor was calculated for snake-VOI and cylinder-VOI for each of the three methods. The relative differences were less than 0.1% (see Additional file 1: Table S2). Thus, the choice of scaling method had negligible effect and provided almost identical results. Thus, we proceed using Method 1 to scale the PBIF to IDIF to generate a sPBIF, because it was simple and required no fitting.

### Clinical validation of PBIF in 171 patients

Clinical patients could have special characteristics (some disease-related) that modulate their circulation and blood-tissue exchange to a degree that undermines the use of a common reference PBIF. Thus, we validated the PBIF for clinical use by quantifying the PBIF-related bias in a group of 171 clinical patients divided into 12 subgroups based on relevant characteristics that could affect the shape their individual IF. For all subgroups, we report the error on AUC (0–60 min) for sPBIF relative to the IDIFs, i.e. an assessment of the error associated with replacing a full 70 min IDIF (obtained from a 70-min multiparametric scan protocol) with an sPBIF scaled to 20 min IDIF from 4 late passes from 50 to 70 min (obtained by a short 20 min multiparametric scan protocol). The relative errors on AUC (0–60 min) introduced by the use of the reference PBIF instead of a 70-min IDIF_Snake_ are shown in Table 4, and in Additional file 1: Table S3 for IDIF_Cyl_. In general, the use of a sPBIF to replace a full IDIF leads to bias less than 5%. For patients with diabetes or low eGFR, the bias was a bit higher at 7%.

**Table 4:** Snake-VOI: errors on AUC (0–60 min) of sPBIF relative to full 70-min IDIF

	Diabetes no	BMI normal	EF normal	BP normal	Age low > 38	eGFR normal
N	20	20	20	20	20	20
R^2^	0.93	0.75	0.96	0.89	0.92	0.95
Bias	2%	4%	4%	3%	1%	1%
SD	4%	6%	5%	7%	6%	5%

	Diabetes yes	BMI high	EF low	BP high	Age high > 75	eGFR low
N	20	20	11	20	20	20
R^2^	0.83	0.88	0.95	0.96	0.92	0.96
Bias	7%	4%	1%	5%	4%	7%
SD	6%	7%	9%	7%	6%	5%

Values are mean ± SD. The results are based on the reference PBIF

### Multiparametric MR_FDG_ imaging using sPBIF

**Fig. 4:**
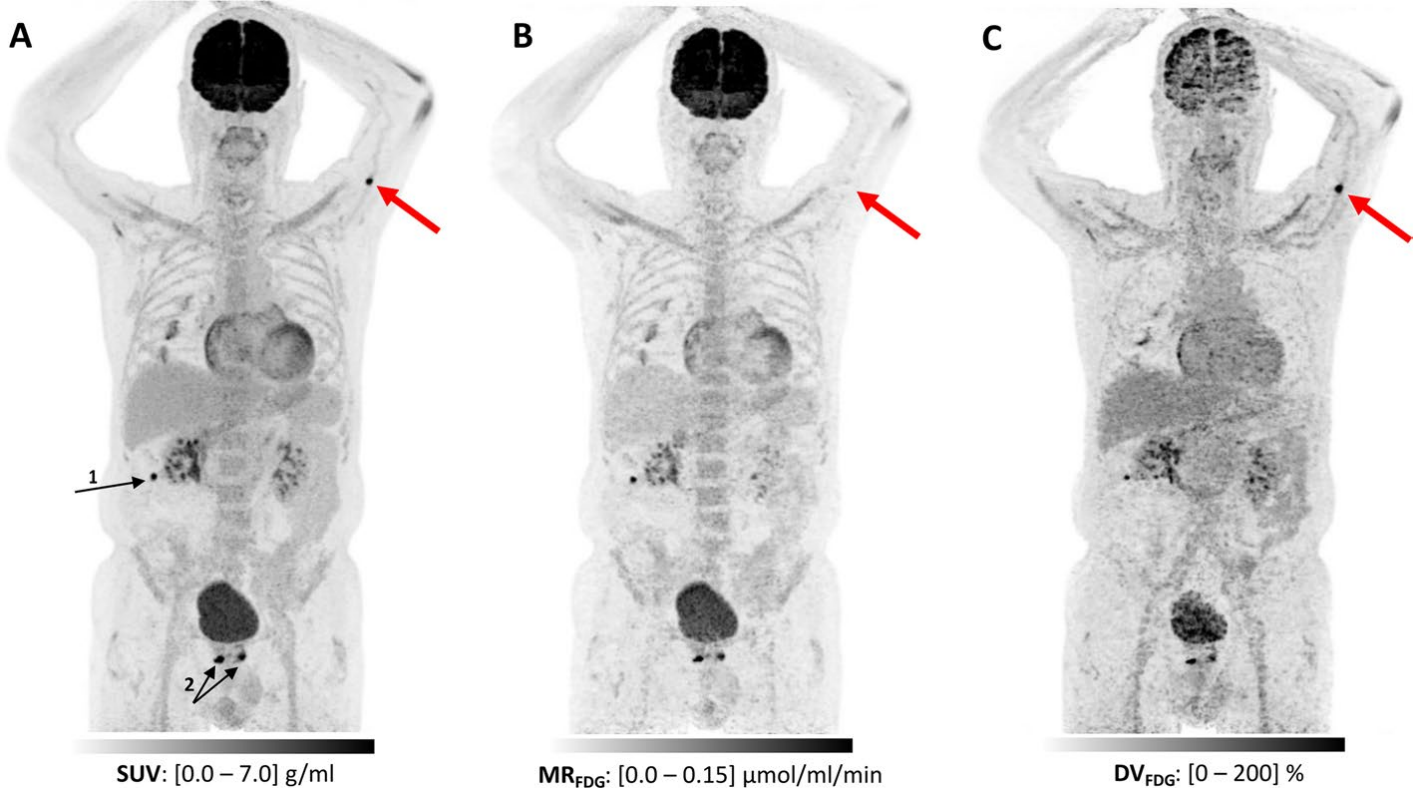
MIPs of the three resulting multiparametric images from a 20-min D-WB examination with four 5 min passes from 50 to 70 min post-injection using PBIF. **A** SUV calculated as the average of all four passes reflecting the total ^18^FDG signal; **B** Patlak MR_FDG_ reflecting the metabolic rate of ^18^FDG, i.e. conversion into ^18^FDG-6P; **C** Patlak DV_FDG_ reflecting the distribution volume of free ^18^FDG. This 85-year-old man was referred for interim evaluation of treatment response after relapse of diffuse large cell lymphoma. SUV images (A), showed regression of nodes on both sides of the diaphragm, but a suspicious newcomer FDG-avid focus was described on the left arm (red arrows), and supplemental localized consolidating radiation treatment was considered. However, further analysis of the Patlak images showed this to be a false-positive finding, as there is no signal on MR_FDG_ images (B), and the presence of signal only on DV_FDG_ images (C) shows this to be unbound FDG. The patient was thus in disease remission and required no supplemental treatment. There were a couple other findings that required investigation. On the right side of the abdomen (black arrow 1) lies a little FDG-avid focus in the colon, in relation to a polyp that on polypectomy showed a high-grade adenoma. There were also bilateral foci in the prostate (black arrows 2). Transrectal ultra-sound could not exclude malignancy, but no biopsy was taken due to the patients' age and comorbidities

**Fig. 5:**
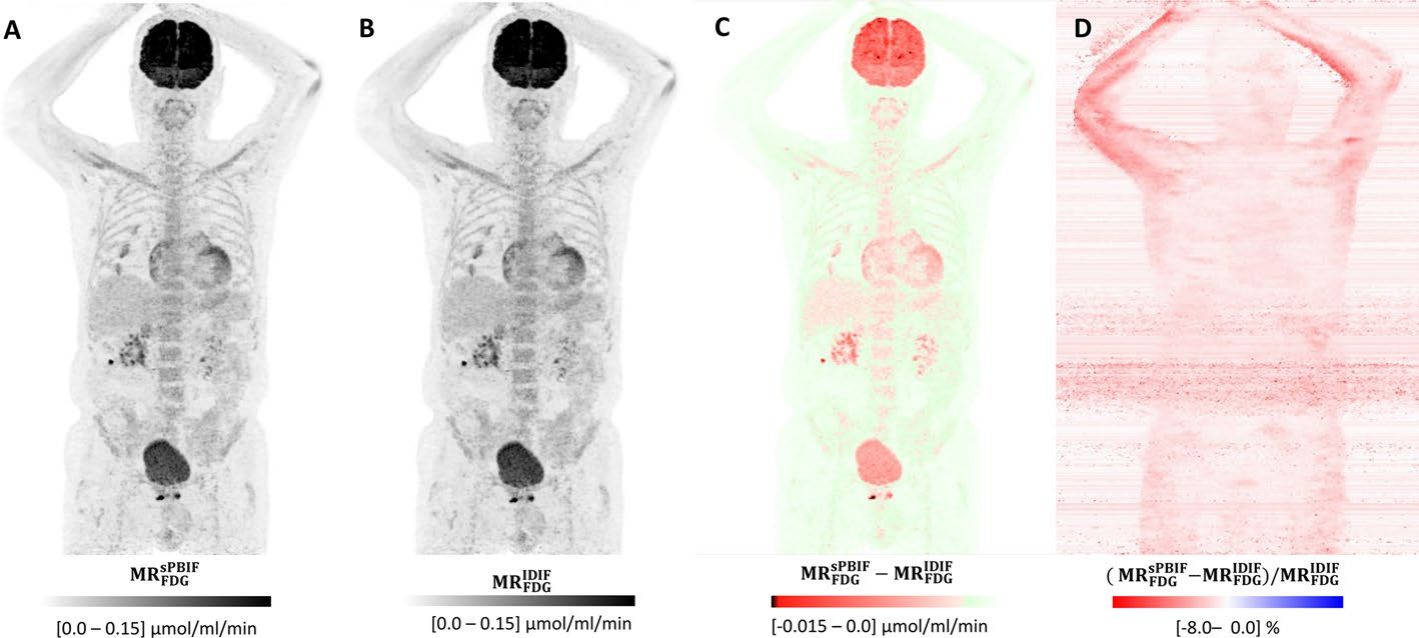
Effect of PBIF illustrated using MIP images of Patlak MR_FDG_ calculated using 4 × 5-min WB passes (50–70 min). **A** MR_FDG_ based on a 20-min D-WB scan (50–70 min) and sPBIF; **B** MR_FDG_ based on a full 70-min IDIF; **C** Absolute difference image; and **D** Relative difference image. The two MR_FDG_ images (**A**) and (**B**) are visually identical. However, the absolute difference image (**C**) shows that the PBIF-based MR_FDG_ image has systematically lower values than the IDIF-based MR_FDG_ image. The relative difference image (**D**) shows almost no anatomical structures, which indicates a constant relative bias. For this patient, the use of sPBIF lead to 4% higher AUC, and consequently to 4% lower MR_FDG_ values. The few structures, e.g. arms, seen on (**D**) are areas with very low MR_FDG_ values. The input function for this patient can be found in Additional file 1: Fig. S5

Figure 4 shows three multiparametric images based on a short 20-min D-WB PET and the reference PBIF, which is the scan protocol shown in Fig. 1C. The MR_FDG_ images are visually indistinguishable from those that would be produced by a long 70-min D-WB PET and IDIF (Fig. 5A, B), but small deviations in AUC between sPBIF and IDIF do lead to small systematic relative errors on MR_FDG_ (Fig. 5C, D). The figure shows organs with a mix of irreversible and reversible kinetics, as well as tissues/lesions with very different MR_FDG_ values. Still, in Fig. 5D, all structures disappear in the subtracted image, i.e. the AUC bias translates directly into a uniform MR_FDG_ bias in all tissue types. The patient has + 4% bias on AUC using sPBIF, which leads to a systematic − 4% bias on the MR_FDG_ images. This way the PBIF-related AUC errors translates into systematic errors on MR_FDG_ image. As seen in Table 4, this AUC bias is usually less than 5%.

## Discussion

Contemporary PET/CT systems can generate more quantitative information about FDG uptake than the SUV by automatically generating Patlak derived parametric images of the metabolic rate and the apparent distribution volume of FDG (MR_FDG_ and DV_FDG_, respectively). The multiparametric PET protocol involves a D-WB examination either performed as a dynamic scan using Total-Body PET scanners or as multiple WB passes using a standard PET/CT with limited axial field-of-view. A major limitation for the clinical adoption of multiparametric FDG imaging is the protracted scan time required to obtain a full IDIF from injection to the end of the PET scan, which is required for Patlak imaging [3, 5]. This limitation may be circumvented by using a sPBIF assuming that the shape of the individual patient’s IF will resemble that of a population’s average IF, and for Patlak analysis, it is particularly important that the AUCs are similar. If this is the case, multiparametric imaging may be based on a short 20 min D-WB PET scan capturing only individual late time point tissue TACs. The shortened imaging time would also reduce the risk of patient motion during the PET scan and potentially improve image quality. Thus, we explored whether substitution of an individual IF by a non-invasive sPBIF results in quantitative bias and errors in the MR_FDG_ images.

It has previously been reported that arterial plasma FDG input functions can be described by relatively simple models. A study [11] recently fitted 90-min arterial samples to a three-exponential model (Eq. 3) and reported that the final dominant exponential was *λ*_3_ = 0.012 min^−1^. Fitting our data to the same model yielded comparable *λ*_3_ = 0.013 min^−1^ and *λ*_3_ = 0.010 min^−1^ for 70 min and 120 min data, respectively. We fitted whole blood arterial blood samples from 0 to 120 min, which is a longer study protocol than what has been used in other studies, and we found that the PBIF was better fitted using a model with four exponentials (Eq. 4) (Table 2). Importantly, the use of more data and a more flexible/advanced model led to better fits to the late part on the IF, which is particularly important for PBIF scaling. In fact, when using our reference PBIF, we found the bias to be within 1% at all time points up to 120 min, which indicates that this PBIF can be used for D-WB imaging at any time window up to at least 2 h (Table 3).

IDIFs were extracted from VOIs placed in the aorta using the CT image and based on automated finding of anatomical landmarks. We also attempted to use IDIFs from the cardiac LV, but initial observations showed that the LV VOIs were affected by partial volume effects and more often needed re-adjustment. Thus, we decided to use aorta VOIs. In our hands, the automated landmarking method proved to be very robust with a success rate of 99%. However, patient motion occurring after acquisition of the CT and before the D-WB PET occasionally caused the CT-based VOI to be slightly off-centre on the D-WB PET, and we therefore recommend to also visually verify the placement of the aortic VOI. This is particularly important for the larger cylinder-VOI, which is more prone to partial volume effect if the VOI centring is not checked. In our study, manual correction was performed in 3 out of 191 patients. On PET/CT systems where the automated landmarking technology is not available, a manually defined and well-centred aorta VOI can be used to extract the aorta IDIF.

We found excellent agreement between the aorta IDIF and the invasive AIF at all times up to 120 min. Thus, it is our opinion that the individual AIF or IDIF can be substituted by a sPBIF using our 120 min PBIF. In contrast, other studies have reported that partial volume effects can lead to systematic errors in which the early part of the IDIF is underestimated, and the late part of the IDIF is overestimated [11, 24]. That we did not observe this is likely explained by the improved spatial resolution of newer PET/CT systems and corresponding reduced partial volume effects. Further underscoring this notion, we found that the IDIFs and AUCs were almost identical using two different aorta VOIs with varying diameters (cylinder-VOI with radius 5 mm; snake-VOI with a single central voxel).

In order to be clinically relevant, the PBIF must be applicable to patients with a wide range of different physiological characteristics and pathologies. For example, the initial peak of the IF could be lower and broader in patients with heart failure or hypertension [25–27], whereas the late part of the IF may be elevated in patients with reduced kidney function or diabetes resulting in decreased excretion of radiotracer [28–30]. To test whether the PBIF was applicable to patients with varying characteristics, we recruited 171 patients (Test group) and did an extensive validation of the PBIF. Overall, we found an acceptable bias less than 5% (Table 4) with the largest bias (7%) observed in patients with diabetes and/or reduced kidney function, whereas the bias was almost negligible in patients with cardiovascular diseases. This can be ascribed to the fact that the overall IDIF AUC is not affected by the shape of the initial peak (as in reduced ventricular ejection fraction) whereas persistently elevated radiotracer (as in kidney failure) results in an increased AUC. We are not aware of any other research teams that have attempted to quantify the impact of disease status on PBIFs, but are of the opinion that a bias of the observed magnitude is acceptable. If not, the estimated MR_FDG_ values could simply be multiplied by a small correction factor (as derived from Table 4). Based on the findings presented here, we have now replaced the extended 70-min D-WB FDG PET protocol (Fig. 1B) with a short 20-min D-WB FDG PET protocol using the scaled PBIF (Fig. 1C) for multiparametric imaging (Fig. 4).

Ideally, the IF for kinetic modelling should be based on plasma FDG activity concentration representing the radiotracer available for uptake in tissue (as opposed to that residing within the erythrocytes). Consequently, Naganawa et al. [11] calculated the PBIF using arterial plasma samples. However, that method does not allow using a short scaled PBIF and the late aorta IDIF, since image-derived blood activity clearly represent whole blood activity, not plasma activity. To address this, Naganawa suggested to scale the PBIF using the initial distribution volume (iDV) which is a function of the patient’s height, weight, and injected dose. The advantage of that approach is that no IDIF is needed. However, the method does not take day-to-day variations of the IF into account, and certain physiological variations are also not contained within the iDV parameter. In addition, the iDV method relies on the injected dose and therefore on dose calibrator cross-calibration and a successful radiotracer injection. We are therefore of the opinion that optimal scaling of the PBIF is preferably done using available D-WB image data, which reflect e.g. extravasation of tracer during injection, reduced kidney function or high blood glucose. Underscoring the robustness of the image-derived approach, we tested three different scaling methods that all produced almost identical results. A further advantage of image-derived scaling is that it is not dependent on dose calibrator or well counter cross-calibrations. The most important caveat of image-derived scaling is probably that the method does not allow for individual plasma to whole blood ratio corrections without resorting to blood sampling and subsequent haematocrit measurement. However, for FDG, the effect of haematocrit correction is quite small and often ignored [31, 32] although it has been demonstrated that the plasma to whole blood ratio for FDG is slightly decreasing as a function of time [11, 33, 34]. Since FDG activity is slightly higher in plasma than in whole blood, correcting for plasma to whole blood ratio would result in a marginally increased AUC of the IF and therefore also lower (on average around 3%) MR_FDG_ values.

## Conclusion

We have demonstrated that a scaled population-based input function (sPBIF) obviates the need for the early D-WB PET acquisition for multiparametric imaging. Thus, imaging of the metabolic rate of FDG, MR_FDG_, can be based on a clinically feasible 20-min D-WB PET protocol without compromising either visual image quality or precision of quantitative PET metrics. The PBIF method can be used with total-body PET scanners or limited axial field-of-view PET scanners employing multiple WB passes. The short D-WB PET examination may be used in clinical routine for a wide range of patients, potentially allowing for more precise quantification in, for example, treatment response imaging.

## Supplementary Information

**Additional file 1**: Paper supplemental Figs. S1, S2, S3, S4, and S5 and supplemental Tables S1, S2, and S3.

**Additional file 2**: The 120-min data PBIF based on Eq.2 + Eq.4. The PBIF was calculated using the parameters in Table 2 (last column) and convoluted with the shape of the MedRad Intego injection. The AUC 0-60 is 100, and this PBIF needs to be scaled to late arterial blood data (blood samples or IDIF) before it is used for parametric imaging. This is the PBIF that we use for our 20-min multiparametric PET protocol.

**Additional file 3**: The same PBIF calculated with Eq.4 and parameters from Table 2 (last column). If needed, readers can convolve this file with the shape of their own injector system.

## Data Availability

Within the restrictions applied by the EU GDPR, all data including the PBIF are available from the authors upon reasonable request.

## Abbreviations

AIC: Akaike information criterion
AIF: Arterial input function
AUC: Area under curve
BMI: Body mass index
BP: Blood pressure
DV_FDG_: Distribution volume of FDG
D-WB: Dynamic whole-body
EF: Ejection fraction (cardiac)
eGFR: Estimated glomerular filtration rate
FDG: ^18^F-fluorodeoxyglucose
IDIF: Image-derived input function
IF: Input function
MR_FDG_: Metabolic rate of FDG uptake
PET/CT: Positron emission tomography/computer tomography
PBIF: Population-based input function
sPBIF: Scaled population-based input function
QC: Quality control
SUV: Standard uptake value
TACs: Time–activity curves
VOIs: Volumes of interest
