# Supplementary material for: Clinical validation of a population-based input function for 20-min dynamic whole-body 18F-FDG multiparametric PET imaging
Source: EJNMMI Phys. 2022 Sep 8;9:60. doi: 10.1186/s40658-022-00490-y (PMC9458803; doi:10.1186/s40658-022-00490-y)
Supplement: Supplementary file 1 — Additional file 1: Paper supplemental Figs. S1, S2, S3, S4, and S5 and supplemental Tables S1, S2, and S3. [file 40658_2022_490_MOESM1_ESM.pdf]

# SUPPLEMENTAL FIGURES

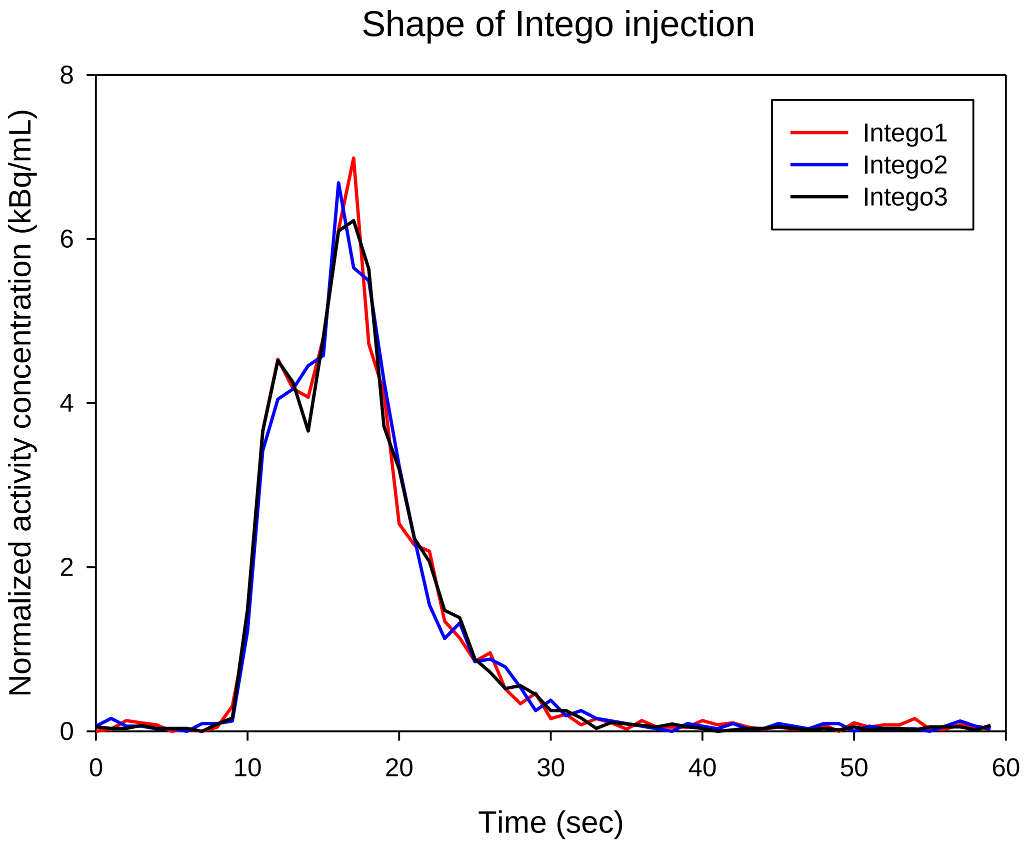


***Supplemental Figure 1.*** *The output shape B(t) from the Intego PET Infusion System (MEDRAD, Inc., Warrendale, PA, USA) measured in three different devices. Activity was withdrawn in the afternoon and infused directly through an Allogg automatic blood sampler next morning. The time-activity curves are normalized to have the same area-under-the-curve. The* $^{18}$*FDG was infused (0.1-3.0 mL; usually 0.1 mL) and flushed with saline (total volume of* $^{18}$*FDG solution and saline is 35 mL). The injection speed is 1 mL/sec, i.e. the injection takes 35-38 sec. The bolus duration was approx. 20 sec (8 sec FWHM).*

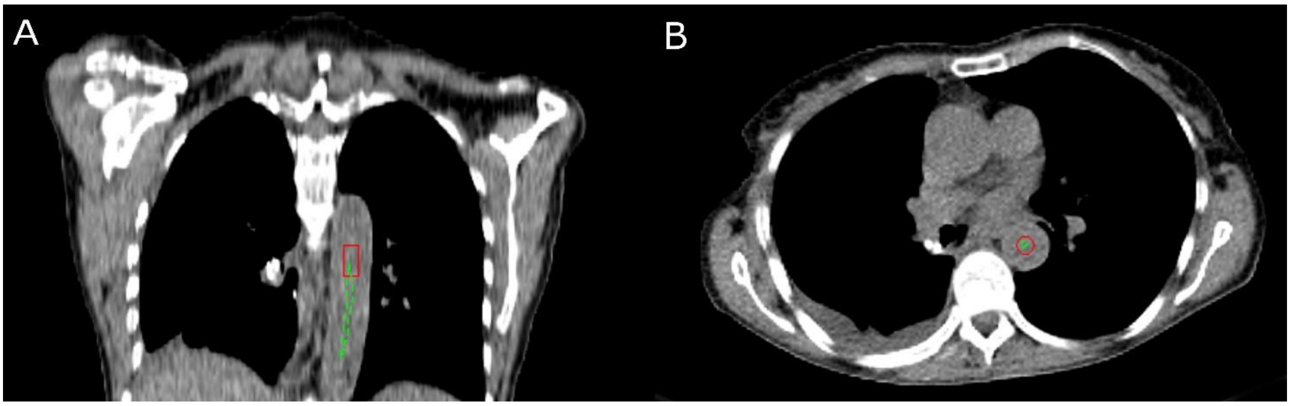


***Supplemental Figure 2.*** *An example from the AIF Group showing the automatically generated cylinder-VOI (red) and the snake-VOI (green). Notice in the coronal-slice (A) and axial-slice (B) that the cylinder-VOI consists of a wider diameter but shorter length, while the snake-VOI has a smaller diameter but a longer length (the entire length of the VOI could not be displayed in a single coronal frame.*

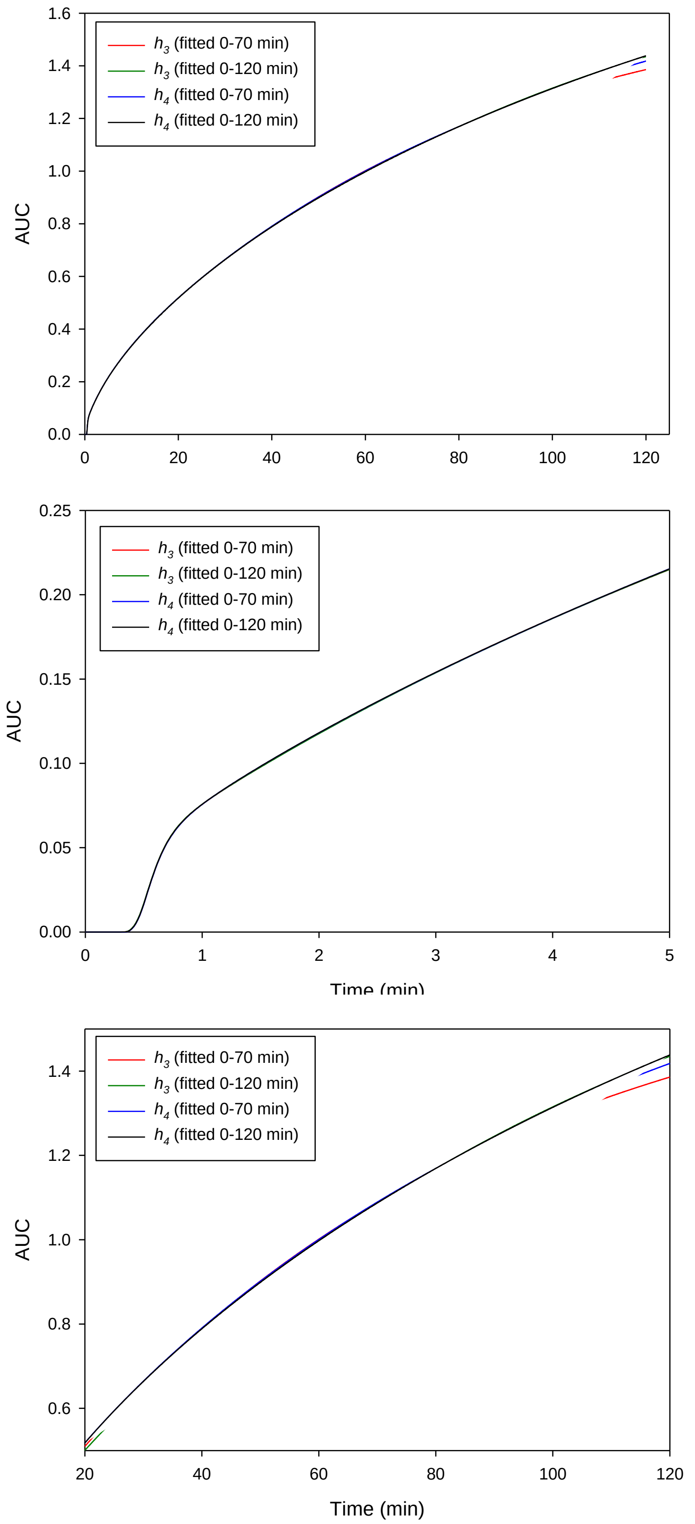


***Supplemental Figure 3:*** *Three plots of AUC of the four PBIF as function of time. The AUC is important as it affects the* $MR_{FDG}$ *estimates.* ***Top:*** *The AUCs are quite similar with small deviations around 40 min and 120 min.* ***Middle:*** *The AUC are completely overlapping during the early dynamic phase following the tracer infusion.* ***Bottom:*** *Focus on the deviations around 40 min and after 90 min.*

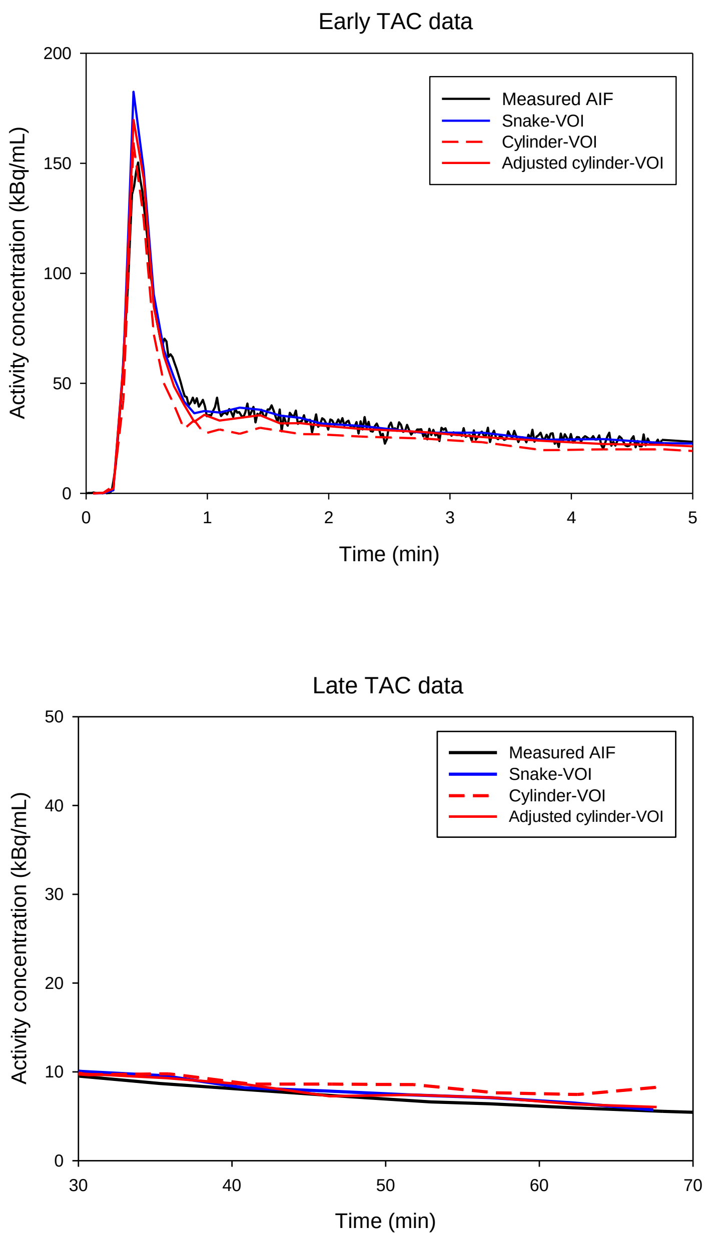


***Supplemental Figure 4.*** *An example from the AIF Group where the automatically generated cylinder-VOI needed a small adjustment. After adjustment there was excellent correlation between AIF and IDIF$_{cyl}$ was restored at early times (top) and late times (bottom). The IDIF$_{snake}$ is shown for comparison.*

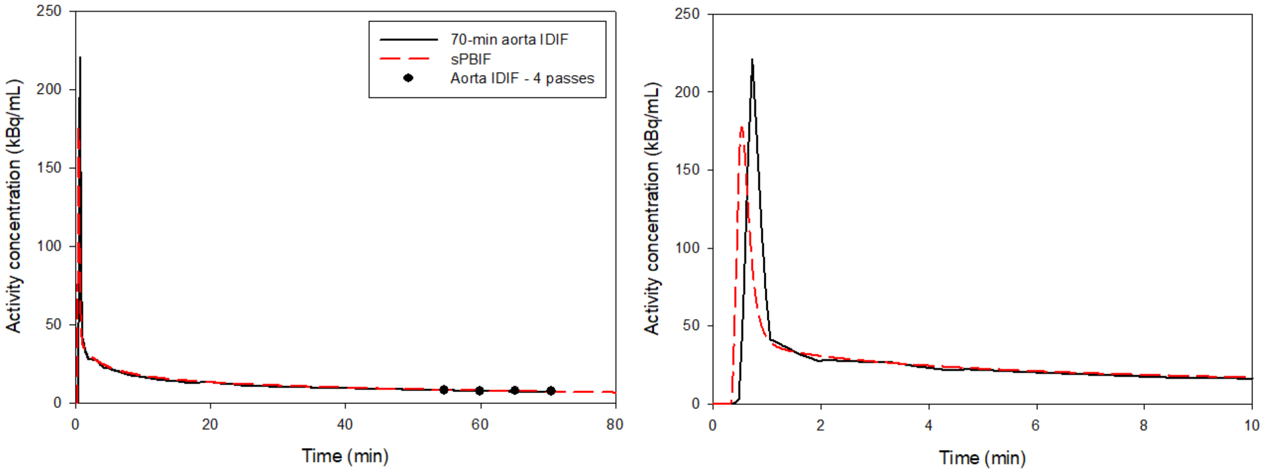


***Supplemental Figure 5.*** *The input function used for the patient in Figure 5.* ***Left:*** *Input function shown from 0 – 70 min;* ***Right:*** *Same data with focus on the early 10 min. For this patient, the IDIF in aorta (as measured using the 70-min scan protocol B) rises slightly later than the PBIF. This later arrival time in aorta is clearly seen on the IDIF from a standard 70-min D-WB PET scan (Scan Protocol B in Figure 1), but it cannot be compensated for with only four late IDIF measurements from a short 20-min scan protocol (Scan Protocol C in Figure 1). Also, close inspection shows that sPBIF is slightly higher that IDIF up to around 40 min. For this patient, the area-under-the-curve is 4% greater for the sPBIF than for the IDIF, which translates into a 4% underestimated* $MR_{FDG}$ *values as seen in Figure 5.*

## SUPPLEMENTAL TABLES

**TABLE 1A: Test-groups: PET study indication and population demographics**

| | Patients with diabetes | | | | | | Patients without diabetes | | | | | |
|---|---|---|---|---|---|---|---|---|---|---|---|---|
| | Number of patients | | | Age distribution* | | | Number of patients | | | Age distribution* | | |
| **Scan indication** | **Total** | **Male** | **Female** | **Total** | **Male** | **Female** | **Total** | **Male** | **Female** | **Total** | **Male** | **Female** |
| Breast cancer | 0 | 0 | 0 | --- | --- | --- | 2 | 0 | 2 | 42 [42-42] | --- | 42 [42-42] |
| Cancer of unknown primary origin | 1 | 1 | 0 | 75 | 75 | --- | 1 | 0 | 1 | 46 | --- | 46 |
| Gastro-intestinal cancer | 1 | 0 | 1 | 57 | -- | 57 | 1 | 1 | 0 | 59 | 59 | --- |
| Gynecological cancer | 1 | 0 | 1 | 63 | --- | 63 | --- | --- | --- | --- | --- | --- |
| Head & Neck cancer | 5 | 3 | 2 | 66.8 [55-77] | 69.7 [62-77] | 62.5 [55-70] | 3 | 2 | 1 | 45.3 [23-70] | 33 [23-43] | 70 |
| Infection & Inflammation | 4 | 3 | 1 | 57.3 [51-74] | 59.3 [52-64] | 51 | 4 | 2 | 2 | 56.3 [46-76] | 46.5 [46-47] | 66 [56-76] |
| Lung cancer | 4 | 3 | 1 | 72.5 [69-77] | 73.7 [72-77] | 69 | 7 | 5 | 2 | 61.4 [47-77] | 61.4 [47-77] | 61.5 [60-63] |
| Lymphoma | 3 | 3 | 0 | 51.7 [28-64] | 51.7 [28-64] | --- | 2 | 1 | 1 | 37 [31-43] | 43 | 31 |
| Urologic cancer | 1 | 1 | 0 | 75 | 75 | --- | -- | -- | -- | --- | --- | --- |
| Total | 20 | 14 | 6 | 63.9 [28-77] | 65.2 [28-77] | 60.8 [51-74] | 20 | 11 | 9 | 52.7 [23-77] | 51.6 [23-77] | 54 [31-76] |

*Values presented as mean[min-max]

**TABLE 1B: Test-groups: PET study indication and population demographics**

| | Patients with EF <50%† | | | | | | Patients with EF ≥ 60% | | | | | |
|---|---|---|---|---|---|---|---|---|---|---|---|---|
| | Number of patients | | | Age distribution* | | | Number of patients | | | Age distribution* | | |
| **Scan indication** | **Total** | **Male** | **Female** | **Total** | **Male** | **Female** | **Total** | **Male** | **Female** | **Total** | **Male** | **Female** |
| Breast cancer | 1 | 0 | 1 | 71 | --- | 71 | 0 | 0 | 0 | --- | --- | --- |
| Cancer of unknown primary origin | 1 | 0 | 1 | 69 | --- | 69 | 1 | --- | 1 | 48 | --- | 48 |
| Gastro-intestinal cancer | 1 | 1 | 0 | 62 | 62 | --- | 2 | --- | 2 | 51 [35-67] | --- | 51 [35-67] |
| Head & Neck cancer | 1 | 1 | 0 | 78 | 78 | --- | 2 | 1 | 1 | 63.5 [59-68] | 68 | 59 |
| Infection & Inflammation | 4 | 4 | 0 | 62.3 [50-82] | 62.3 [50-82] | --- | 6 | 4 | 2 | 49.3 [22-73] | 46.8 [22-73] | 54.5 [53-56] |
| Lung cancer | 2 | 2 | 0 | 73 [72-74] | 73 [72-74] | --- | 7 | 4 | 3 | 66.1 [50-81] | 70.8 [50-81] | 60 [58-62] |
| Lymphoma | 1 | 1 | 0 | 85 | 85 | --- | 2 | 1 | 1 | 74 [72-76] | 76 | 72 |
| Total | 11 | 9 | 2 | 69.5 [50-85] | 68.9 [50-85] | 70 [69-71] | 20 | 9 | 11 | 59.2 [22-81] | 61.4 [22-81] | 57.0 [35-72] |

*Values presented as mean [min-max]
† EF values evaluated by echocardiography [min-max] = [30-45%]

| TABLE 1C: Test-groups: PET study indication and population demographics | | | | | | | | | | | | |
|---|---|---|---|---|---|---|---|---|---|---|---|---|
| | **Patients with high BP (>140)** | | | | | | **Patients with normal BP (<120/80)** | | | | | |
| | **Number of patients** | | | **Age distribution*** | | | **Number of patients** | | | **Age distribution*** | | |
| **Scan indication** | **Total** | **Male** | **Female** | **Total** | **Male** | **Female** | **Total** | **Male** | **Female** | **Total** | **Male** | **Female** |
| Breast cancer | 1 | 0 | 1 | 84 | --- | 84 | 2 | --- | 2 | 46.5 [42-51] | --- | 46.5 [42-51] |
| Cancer of unknown primary origin | 0 | 0 | 0 | --- | --- | --- | 1 | 1 | --- | 38 | 38 | --- |
| Head & Neck cancer | 7 | 5 | 2 | 60.7 [31-70] | 59 [31-70] | 65 [63-67] | 1 | 1 | --- | 54 | 54 | --- |
| Infection & Inflammation | 4 | 1 | 3 | 53.8 [38-75] | 51 | 54.7 [38-75] | 8 | 4 | 4 | 56.6 [23-75] | 65.5 [55-75] | 47.8 [23-59] |
| Lung cancer | 0 | 0 | 0 | --- | --- | --- | 7 | 3 | 4 | 66 [58-81] | 74.3 [66-81] | 59.8 [58-62] |
| Lymphoma | 0 | 0 | 0 | --- | --- | --- | 1 | 0 | 1 | 31 | --- | 31 |
| Total | 20 | 11 | 9 | 61.6 [31-84] | 58.9 [31-70] | 64.9 [38-84] | 20 | 10 | 10 | 56.6 [23-81] | 65.4 [38-81] | 51.1 [23-61] |

*Values presented as mean [min-max]

| TABLE 1D: Test-groups: PET study indication and population demographics | | | | | | | | | | | | |
|---|---|---|---|---|---|---|---|---|---|---|---|---|
| | **Patients with BMI (>30)†** | | | | | | **Patients with normal BMI (20-22)†** | | | | | |
| | **Number of patients** | | | **Age distribution*** | | | **Number of patients** | | | **Age distribution*** | | |
| **Scan indication** | **Total** | **Male** | **Female** | **Total** | **Male** | **Female** | **Total** | **Male** | **Female** | **Total** | **Male** | **Female** |
| Breast cancer | 1 | --- | 1 | 65 | --- | 65 | --- | --- | --- | --- | --- | --- |
| Cancer of unknown primary origin | --- | --- | --- | --- | --- | --- | 3 | 1 | 2 | 65.3 [64-68] | 68 | 64 [64-64] |
| Head & Neck cancer | 3 | 3 | --- | 64.3 [51-70] | 64.3 [51-70] | --- | 5 | 1 | 4 | 58.6 [40-75] | 57 | 59 [40-75] |
| Infection & Inflammation | 11 | 5 | 6 | 49.5 [32-75] | 46.8 [34-62] | 51.8 [32-75] | 3 | 1 | 1 | 56 [53-59] | 59 | 53 |
| Lung cancer | 3 | 1 | 2 | 63.3 [54-74] | 74 | 58 [54-62] | 7 | 2 | 5 | 65.6 [51-77] | 76.5 [76-77] | 61.2 [51-67] |
| Lymphoma | 2 | 1 | 1 | 33 [28-37] | 28 | 37 | 1 | --- | 1 | 60 | --- | 60 |
| NET | --- | --- | --- | --- | --- | --- | 1 | --- | 1 | 69 | --- | 69 |
| Total | 10 | 10 | 10 | 52.9 [28-75] | 52.9 [28-74] | 52.9 [32-75] | 20 | 5 | 15 | 62.8 [40-77] | 67.4 [57-77] | 61.3 [40-75] |

*Values presented as mean [min-max]
† Patients with BMI >30: mean BMI=35.8 [32.3-45.5]; Patients with normal BMI (20-22): mean BMI=20.97 [20.2-22]

| TABLE 1E: Test-groups: PET study indication and population demographics | | | | | | | | | | | | |
|---|---|---|---|---|---|---|---|---|---|---|---|---|
| | **Patients < 38 years old** | | | | | | **Patients > 75 years old** | | | | | |
| | **Number of patients** | | | **Age distribution*** | | | **Number of patients** | | | **Age distribution*** | | |
| **Scan indication** | **Total** | **Male** | **Female** | **Total** | **Male** | **Female** | **Total** | **Male** | **Female** | **Total** | **Male** | **Female** |
| Breast cancer | 0 | 0 | 0 | --- | --- | --- | 2 | 0 | 2 | 80 [77-83] | --- | 80 [77-83] |
| Cancer of unknown primary origin | 1 | 0 | 1 | 35 | --- | 35 | 1 | 1 | 0 | 78 | 78 | --- |
| Gastro-intestinal cancer | 1 | 0 | 1 | 35 | --- | 35 | 0 | 0 | 0 | --- | --- | --- |
| Head & Neck cancer | 2 | 2 | 0 | 27 [23-31] | 27 [23-31] | --- | 1 | 1 | 0 | 81 | 81 | --- |
| Infection & Inflammation | 9 | 4 | 5 | 29 [22-37] | 30.8 [22-34] | 27.6 [22-37] | 4 | 3 | 1 | 77.8 [76-82] | 78.3 [76-82] | 76 |
| Lung cancer | 0 | 0 | 0 | --- | --- | --- | 10 | 8 | 2 | 79.4 [76-83] | 79.3 [76-83] | 80 [79-81] |
| Lymphoma | 6 | 3 | 3 | 27.5 [18-37] | 25 [18-29] | 30 [22-37] | 2 | 2 | 0 | 77 [77-77] | 77 [77-77] | --- |
| NET | 1 | 1 | 0 | 30 | 30 | --- | 0 | 0 | 0 | --- | --- | --- |
| Total | 20 | 10 | 10 | 29 [18-37] | 28.2 [18-34] | 29.8 [22-37] | 20 | 15 | 5 | 78.9 [76-83] | 78.8 [76-83] | 79.2 [76-83] |
| *Values presented as mean[min-max] | | | | | | | | | | | | |

| TABLE 1F: Test-groups: PET study indication and population demographics | | | | | | | | | | | | |
|---|---|---|---|---|---|---|---|---|---|---|---|---|
| | **Patients with low eGFR (<60)†** | | | | | | **Patients with normal eGFR (>90)** | | | | | |
| | **Number of patients** | | | **Age distribution*** | | | **Number of patients** | | | **Age distribution*** | | |
| **Scan indication** | **Total** | **Male** | **Female** | **Total** | **Male** | **Female** | **Total** | **Male** | **Female** | **Total** | **Male** | **Female** |
| Breast cancer | 1 | 0 | 1 | 83 | --- | 83 | 0 | 0 | 0 | --- | --- | --- |
| Cancer of unknown primary origin | 1 | 1 | 0 | 78 | 78 | --- | 3 | 1 | 2 | 50.7 [35-64] | 53 | 49.5 [35-64] |
| Head & Neck cancer | 3 | 1 | 2 | 70.3 [55-81] | 81 | 65 [55-75] | 5 | 2 | 3 | 51.4 [41-61] | 46.5 [41-52] | 54.7 [49-61] |
| Infection & Inflammation | 2 | 2 | 0 | 76.5 [71-82] | 76.5 [71-82] | --- | 4 | 2 | 2 | 47.5 [22-61] | 40.5 [22-59] | 54.5 [48-61] |
| Lung cancer | 8 | 6 | 2 | 73.5 [66-83] | 75.8 [67-83] | 66.5 [66-67] | 6 | 3 | 3 | 56.5 [49-62] | 52.7 [49-59] | 60.3 [58-62] |
| Lymphoma | 3 | 3 | 0 | 76.3 [67-85] | 76.3 [67-85] | --- | 2 | 2 | 0 | 36 [29-43] | 36 [29-43] | --- |
| NET | 1 | 1 | 0 | 30 | 30 | --- | 0 | 0 | 0 | --- | --- | --- |
| Urogenital cancer | 1 | 1 | 0 | 75 | 75 | --- | 0 | 0 | 0 | --- | --- | --- |
| Total | 20 | 15 | 5 | 82.4 [30-85] | 73.4 [30-85] | 69.2 [55-83] | 20 | 10 | 10 | 50.5 [22-64] | 45.7 [22-59] | 55.3 [35-64] |
| *Values presented as mean [min-max]<br>† Patients with eGFR <60: mean eGFR=48 [19-58] | | | | | | | | | | | | |

**Supplemental Table 2. Relative differences of Scale Factors**

| | Method 1 | Method 2 | Method 3 |
|---|---|---|---|
| Snake-VOI | -0.05 % ± 0.04 % | 0.02 % ± 0.11 % | 0.03 % ± 0.14 % |
| Cylinder-VOI | -0.03 % ± 0.41 % | 0.05 % ± 0.41 % | -0.01 % ± 0.82 % |

The relative differences of scale factors (SF) were calculated relative to the mean value of all methods, e.g. scale factor for method 1: SF1 relative to (SF1 + SF2 + SF3)/3. Values are mean ± SD (N=20).

**Supplemental Table 3: Cylinder-VOI: Errors on AUC (0-60 min) of sPBIF relative to full 70-min IDIF**

| | **Diabetes No** | **BMI Normal** | **EF Normal** | **BT Normal** | **Age Low > 38** | **eGFR Normal** |
|---|---|---|---|---|---|---|
| N | 20 | 20 | 20 | 20 | 20 | 20 |
| $R^2$ | 0.89 | 0.84 | 0.95 | 0.88 | 0.92 | 0.93 |
| Bias | 4% | 4% | 4% | 4% | 2% | 1% |
| SD | 6% | 4% | 4% | 7% | 7% | 6% |
| | **Diabetes Yes** | **BMI High** | **EF Low** | **BT High** | **Age High > 75** | **eGFR Low** |
| N | 20 | 20 | 11 | 20 | 20 | 20 |
| $R^2$ | 0.86 | 0.87 | 0.95 | 0.94 | 0.92 | 0.95 |
| Bias (%) | 6% | 4% | 1% | 4% | 5% | 8% |
| SD (%) | 7% | 8% | 8% | 7% | 5% | 6% |
